# Naturalistic assessments across the lifespan: Systematic review of inhibition measures in ecological settings

**DOI:** 10.1016/j.neubiorev.2024.105915

**Published:** 2024-12

**Authors:** Larisa-Maria Dina, Tim J. Smith, Tobias U. Hauser, Eleanor J. Dommett

**Affiliations:** a Department of Psychology, King’s College London, London SE1 1UL, United Kingdom; b Centre for Brain and Cognitive Development, Department of Psychological Sciences, Birkbeck College, London WC1E 7HX, United Kingdom; c Creative Computing Institute, University of the Arts, London SE5 8UF, United Kingdom; d Max Planck UCL Centre for Computational Psychiatry and Ageing Research, London WC1B 5EH, United Kingdom; e Wellcome Centre for Human Neuroimaging, University College London, London, UK; f Department of Psychiatry and Psychotherapy, Medical School and University Hospital, Eberhard Karls University of Tübingen, Tübingen, Germany; g German Center for Mental Health (DZPG), Germany

**Keywords:** Inhibitory control, Gamification, Gamified, Virtual reality, Ecological momentary assessment, Ambulatory assessment, Lifespan, Systematic review, Real-world, Technology, Digital health

## Abstract

Inhibitory control is essential for our everyday lives. Despite this, it is commonly assessed using non-naturalistic assessments. In this systematic review, we argue for the importance of taking an ecological approach to assess cognition. The aims are to present the state-of-knowledge in naturalistic assessments of inhibitory control, focusing on their methodological characteristics, including psychometric properties and user experience. PubMed, PsycINFO and Web of Science were searched until September 2024. Studies were included if they used at least one naturalistic method of assessing inhibition. The included studies (N=64) were grouped into three methodological categories: gamification, virtual reality, and brief, repeated assessments in participants’ usual environment in the form of ecological momentary assessments. Sample sizes spanned three orders of magnitude (N=12–22,098). We report considerable heterogeneity in the types of tasks used, and the psychometric details reported. Nonetheless, naturalistic tasks were generally comparable with standardised equivalents, although some tasks assessed mixed-domain constructs. Tasks were feasible and acceptable for participants, with generally high completion rates and engagement. Recommendations for future research are discussed.

## Highlights

• Gamified, virtual reality and ecological momentary tasks were included.
• Considerable heterogeneity in the types of tasks, and psychometric details reported.
• Generally, performance on naturalistic tasks comparable to standardised tasks.
• Naturalistic tasks were feasible and acceptable for most participants.
• Careful development and implementation of naturalistic tasks is needed.

## 1 Introduction

Inhibitory control is a core executive function, commonly seen alongside working memory and cognitive flexibility (Diamond, 2013) (albeit other classifications exist; e.g., Jurado and Rosselli, 2007; Miyake and Friedman, 2012), and refers to the ability to actively suppress or delay responses with the intention of achieving a goal. Inhibitory control is essential for our everyday lives, and impairment is associated with numerous psychiatric disorders, including attention-deficit/hyperactivity disorder, obsessive-compulsive disorder (OCD), anxiety and depression. For example, deficits in inhibitory control might lead individuals with OCD and anxiety to have difficulty changing or stopping habitual and inappropriate thoughts (Fitzgerald et al., 2021, Pan et al., 2023) and might be implicated in suicidal behaviours in affective disorders such as depression (Richard-Devantoy et al., 2012). In cognitive neuroscience, ‘inhibitory control’ is often used as an umbrella term to refer to the multiple facets of inhibition, including cognitive, response and emotional inhibition (Feola et al., 2023). Cognitive inhibition refers to supressing competing cognitive processes to solve problems, response inhibition refers to supressing prepotent responses and replace them with context-appropriate responses, and emotional inhibition refers to the suppression of task-irrelevant emotional information (Hung et al., 2018). The notion that inhibitory control is a multicomponent executive function has been further supported by neuroimaging evidence (Hung et al., 2018). In this review, we use the term inhibitory control to refer to the inhibitory control domain of response inhibition, which primarily activates a fronto-striatal system (Hung et al., 2018).

Despite its importance in everyday behaviours, inhibitory control is typically assessed in non-naturalistic, highly controlled environments such as laboratories. Laboratory tasks filter irrelevant stimuli, which are considered noise or confounds, and aim to isolate specific latent variables, which are considered signal (Nastase et al., 2020). However, in doing so they do not adequately mimic the complexities of everyday life (Munakata et al., 2011). Most ecological situations involve both task relevant and task irrelevant information which require our brains to constantly evaluate and re-evaluate information by considering the context and the goal. This means that classical controlled experiments where most task irrelevant stimuli are filtered out overlook a central challenge our brains are faced with in our everyday lives (Nastase et al., 2020). It has been proposed that an ecological approach to the study of cognition is essential for advancing cognitive science (Henry et al., 2012b), and thus it is important to measure cognition using more naturalistic methods. Here we define naturalistic methods as being on the latter end of a continuum from static, decontextualised, repeated stimuli with low ecological validity to dynamic, contextualised, continuous and often multisensory stimuli with high ecological validity (Aliko et al., 2020). Naturalistic methods, such as games, should also facilitate a level of enjoyment, by increasing intrinsic motivation and, therefore, participant engagement (Allen et al., 2024).

**Fig. 1:**
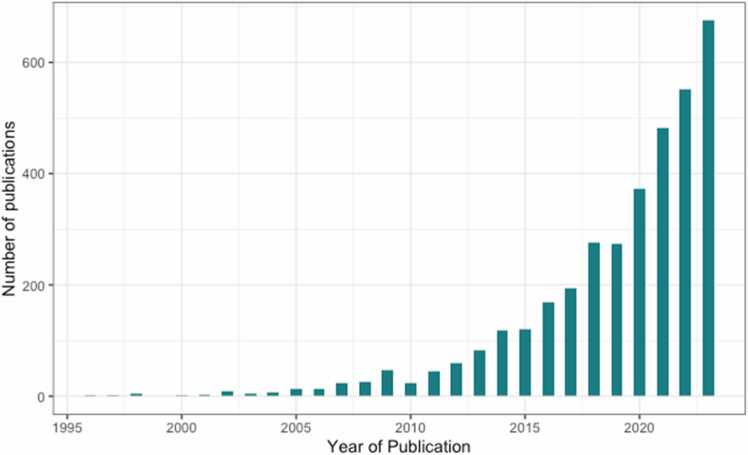
Number of ecological momentary assessment publications by year.

To achieve this, it is possible to either bring more realistic stimuli into the laboratory (isolating latent variables while introducing some curated noise, e.g., through immersive virtual reality environments) or bring the laboratory into the real world (measuring aspects of the environment that might influence cognition, e.g., through ecological sampling methods capable of measuring dynamic, continuous data). The latter approach has increased substantially in recent years with a surge in the number of publications using ecological methods (e.g., ecological momentary assessments; Fig. 1). This two-pronged conceptualisation is further supported by a recently published ecological brain framework which proposes that there should be a cyclicity between naturalistic, real-world exploratory studies and artificial, lab-based confirmatory studies to successfully handle the complexity of ecological approaches to the study of cognition (Vigliocco et al., 2023). The current review uses this framework as a guide to identify task-based, virtual naturalistic assessments of inhibitory control, although it is important to note that these may sit at different points across the axis of naturalism.

In response to the surge in the number of publications using ecological methods (e.g., ecological momentary assessments; Fig. 1) in recent years, the centrality of inhibitory control to our everyday lives, and the considerable heterogeneity in existing inhibitory control tasks, we conducted the first systematic review to assess the characteristics of task-based, virtual naturalistic assessments of inhibitory control. While inhibitory control can be used as an umbrella term to refer to cognitive, response and emotional inhibition (Feola et al., 2023), the current review focuses on response inhibition. Findings from such a review would provide a useful resource for researchers interested in both the development and application of naturalistic paradigms and could help foster collaboration and optimise the use of resources as using such tasks usually require specialist software and hardware, and technical expertise that might not be available in the immediate research teams.

The current systematic review presents the current research using task-based, virtual naturalistic assessments to measure inhibitory control across the lifespan by summarising the methodological features of these naturalistic assessments (e.g., setting, sample characteristics, task characteristics, psychometric properties, user experience). Reviewing 64 studies spanning three methodological modalities (gamified, virtual reality and ecological momentary tasks), we find that naturalistic assessments for inhibitory control are largely comparable to standardised equivalents, and that they are feasible and acceptable to most participants. Nonetheless, as expected, we report considerable heterogeneity in the types of tasks and psychometric details reported. We discuss these findings and their implications for naturalistic cognitive research and digital health.

## 2 Methods

The Preferred Reporting Items for Systematic Reviews and Meta-Analyses (PRISMA) checklist (Page et al., 2021) was used in the design and reporting of this review. The review protocol was submitted and pre-registered on the Open Science Framework (https://osf.io/zshkg/).

### 2.1 Inclusion criteria

This systematic review focused on published studies using naturalistic assessments to measure inhibition across the lifespan. Studies needed to include at least one task-based, virtual naturalistic method of assessing inhibition in the real-world, such as electronic handheld device- or external sensor-assessed inhibition (e.g., through ecological sampling methodologies); or a laboratory assessment of inhibition capturing naturalistic behaviours in a virtual environment (e.g., gamification, virtual reality). The studies had to be available in English and published in a peer-reviewed journal.

### 2.2 Exclusion criteria

We excluded studies focusing on cognitive training methods, since the focus of this review is on naturalistic assessment methodologies rather than naturalistic interventions. Studies were excluded if the full text could not be accessed by the authors. Case studies and conference abstracts were also not included.

### 2.3 Search strategy

PubMed, PsycINFO and Web of Science were initially searched between November 2022 and February 2023. We then conducted an updated search in September 2024 to cover the period between February 2023 and September 2024.We combined three groups of terms to form the search strategy. The first group referred to the population being studied (e.g., infants, toddlers, children, adolescents, adults), the second referred to the methods (e.g., from adjectives such as naturalistic, ecological, real-world to methods such as virtual reality, gamified tasks, functional near infrared spectroscopy), and the third referred to the outcome of interest (inhibitory control). The rationale for including methods such as functional near infrared spectroscopy (fNIRS) in the search was to capture any articles that may use wireless and portable imaging equipment in naturalistic environments (Pinti et al., 2018), such as non-static laboratory settings (e.g., Bulgarelli et al., 2023) or in the real-world (e.g., Burgess et al., 2022). The search was not restricted to a specific timeline, and instead included all eligible studies published until the last search was performed (in September 2024). We hand-searched reference lists of available systematic reviews of naturalistic assessments of executive functions and used the expertise within the review team to identify additional articles of interest. The full search strategy is reported in the Supplementary materials.

### 2.4 Selection of studies

We merged and deduplicated the identified records using a reference manager (EndNote) and Rayyan.ai (Ouzzani et al., 2016). Following deduplication, two reviewers (LD, EJD) independently and blindly screened 10 % (N = 567) of the titles and abstracts of the initial search (up to February 2023)) using Rayyan.ai (include, exclude, maybe. For the full texts screening of the studies identified in the original search, two reviewers (LD, EJD) independently and blindly screened 10 % of the included studies (N = 11) against the pre-specified inclusion criteria (include, exclude, maybe). Potential discrepancies were resolved by discussions with the other authors (TJS, TUH). The rest of the studies in the original search (90 %) were screened by the first author (LD). For the updated search (February 2023 – September 2024), the titles, abstracts and full texts were screened by the first author (LD) in consultation with EJD, TJS and TUH. In accordance with the PRISMA checklist, primary reasons for excluding each study were recorded at the full text screening stage. The reasons for excluding studies were: full text unavailable; study not published in English; study protocol; conference abstract; duplicate; wrong study design (not using naturalistic assessments).

### 2.5 Data extraction and management

A data extraction form was developed in Microsoft Excel by two reviewers (LD and EJD) in collaboration with the larger review team to extract information on study description, participant characteristics, setting, methods of assessment, task characteristics, and comparisons between standardised and naturalistic assessments. The full description of the extracted information is available on the Open Science Framework (https://osf.io/zshkg/).

### 2.6 Quality appraisal

Because no fit-for-purpose quality appraisal tool could be identified for the reporting of naturalistic or real-world methodologies, we decided to use two quality appraisal tools based on the study designs of the included studies. For studies employing an EMA design, a quality appraisal tool developed by Liao et al. (2020) and adapted by Kwasnicka et al. (2021) was used, which included the following four criteria: 1) rationale for EMA design; 2) whether an a priori power analysis had been conducted; 3) adherence to EMA protocol; 4) missingness analysis. The quality of each EMA study was rated as weak, moderate, or strong. For studies using cross-sectional designs, we used the Appraisal tool for Cross-Sectional Studies (AXIS). The table summarising the questions and the ratings of the included studies is presented in Table S1 in the Supplementary Materials and on OSF (https://osf.io/zshkg/).

## 3 Results

**Fig. 2:**
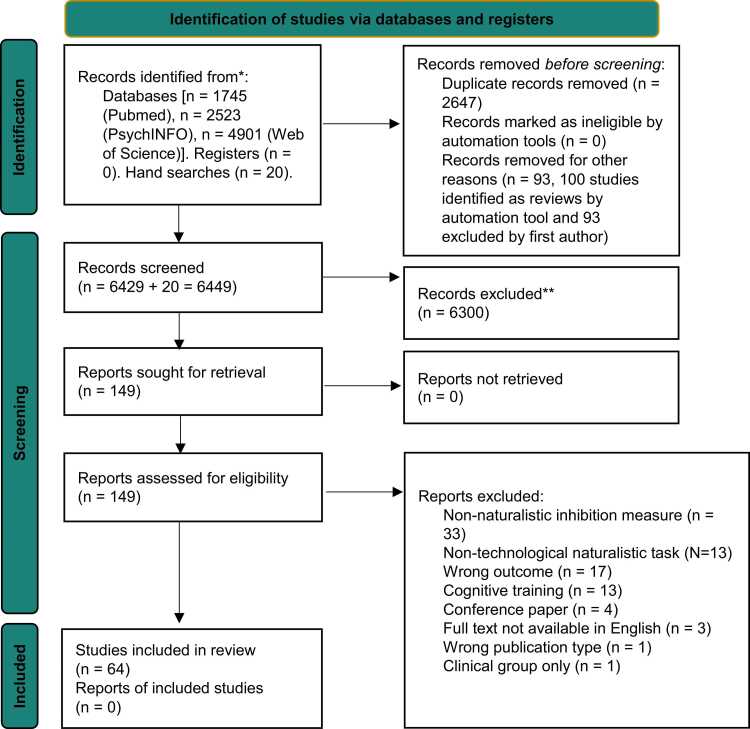
The Preferred Reporting Items for Systematic reviews and Meta-Analyses (PRISMA) flowchart, where we break down the steps taken to identify and screen the studies included in this review.

The original search identified 8002 studies through database searching and 18 through handsearching, of which 2260 studies were removed because they were duplicates and 93 studies were taken out because they were reviews. In the next step, studies were screened by abstract and title. This process excluded 5535 studies, and 132 studies were assessed for eligibility by screening the full text. Of those, 51 studies met the inclusion criteria in the original search and were included in the qualitative synthesis for the systematic review (Fig. 2). The updated search identified 1167 studies through database searching and 2 through handsearching, of which 387 studies were removed because they were duplicates. The title and abstract screening excluded 765 studies, and 17 studies were assessed for eligibility by screening the full text. Of those, 13 studies met the inclusion criteria and were further added to the qualitative synthesis. Therefore, Fig. 2 below shows the combined records identified, screened and included in the original and updated searches (included studies, N = 64).

The included studies were categorised based on the methodological characteristics of the naturalistic inhibitory control task they used. These categories were not decided on *a priori* since we could not know exactly which types of studies we would find. Instead, we grouped the studies into categories after all eligible studies were identified and included in the review (n = 64). The included studies were categorised into gamified tasks (n = 23), virtual reality tasks (n = 30), and ecological momentary assessment tasks (n = 12). One study was included in both the gamified and virtual reality categories (Chicchi Giglioli et al., 2021). Here we define gamified tasks as those that implement features from gaming for non-game purposes (e.g., milestones, competition, rankings, personalisation) (Robson et al., 2015, Sailer et al., 2017), virtual reality tasks as those that involve interactive, immersive and advanced computer technologies to generate a 3D environment (Neguț et al., 2016), and ecological momentary assessment tasks as those that are brief, repeatable and can be self-administered via smartphones or other handheld devices in participants’ usual environment as they go about their day-to-day lives (Singh et al., 2023). Included studies and the tasks are presented in TS3 in the Supplementary Materials.

### 3.1 Gamified inhibition tasks

#### 3.1.1 Country of data collection

The studies were conducted in different countries, including UK (N = 3), Canada (N = 3), Germany (N = 3), Italy (N = 2), Spain (N = 3), Netherlands (N = 1), Sweden (N = 1), Ireland (N = 1), India (N = 1), China (N = 1), Chile (N = 1), Switzerland (N = 1), Argentina (N = 1), Uruguay (N = 1), and Australia (N = 1). To note, one study was conducted across three countries (Argentina, Uruguay and Spain) (Vladisauskas et al., 2024).

#### 3.1.2 Participant characteristics

Sample sizes ranged from 16 to 22,098 participants (median = 83, IQR = 76.5), with a total of 24,973 participants. Overall, 24,821 typically developing (median = 69, IQR = 65.5), 109 individuals with ADHD (M = 27.25, SD = 25.55) and 43 individuals with intellectual disabilities were included in this review. For typically developing individuals, ages ranged from 3 to 66 years. Overall, typically developing individuals had a mean age of 19.91 (SD = 15.96). Female participants comprised 45 % of the overall sample. Regarding developmental stages, four studies included pre-school children (Axelsson et al., 2016, Bhavnani et al., 2019, Delgado et al., 2016, Peijnenborgh et al., 2016), eleven studies included elementary school children (Brkic et al., 2022, Crepaldi et al., 2020a, Crepaldi et al., 2020b, Delgado et al., 2016, Gallagher et al., 2023, Heemskerk and Roebers, 2023, Johann and Karbach, 2018, Lawrence et al., 2002, Peijnenborgh et al., 2016, Rivero et al., 2021, Vladisauskas et al., 2024), nine included adults (Chicchi Giglioli et al., 2018, Chicchi Giglioli et al., 2021, Friehs et al., 2020, Friehs et al., 2021, Friehs et al., 2022, Lumsden et al., 2017, Schroeder et al., 2021, Smittenaar et al., 2015, Tong et al., 2021), and one study included older adults (Wang et al., 2023). Some studies did not report the mean age of participants (Smittenaar et al., 2015), the exact age ranges (Chicchi Giglioli et al., 2021, Friehs et al., 2022, Lumsden et al., 2017, Schroeder et al., 2021, Smittenaar et al., 2015) nor the gender split of the sample (Delgado et al., 2016, Smittenaar et al., 2015) and these are not included in the calculations.

#### 3.1.3 Types of tasks

**Fig. 3:**
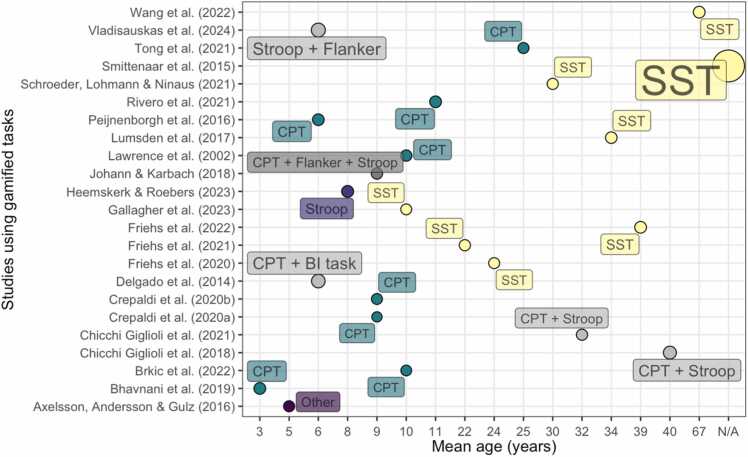
Tasks used in the gamification category and mean age (years, rounded) of the samples tested. The size of the circles provides an approximate estimation of sample size (i.e., a larger size indicates a larger sample size).

Tasks are summarised in Fig. 3. Most studies used gamified versions of a continuous performance task (CPT) (N = 11, 48 %), followed by stop-signal tasks (SST) (N = 8, 35 %), Stroop tasks (N = 4, 17 %), a Wizard-of-Oz implementation (N = 1, 4 %), a Flanker task (N = 1, 4 %) and a behavioural inhibition task (N = 1, 4 %).

#### 3.1.4 Psychometric properties of the gamified tasks

**Fig. 4:**
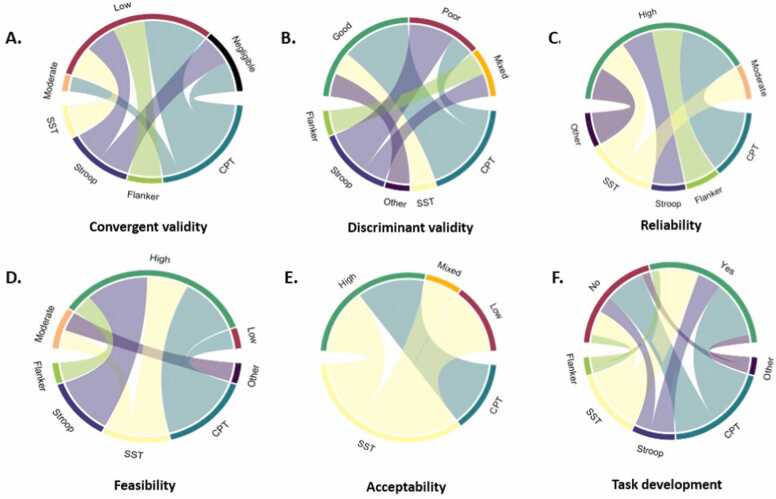
The psychometric characteristics of the various task types in the gamification category: (A) convergent validity, (B) discriminant validity, (C) reliability, (D) feasibility, (E) acceptability, (F) provided information on task development (yes/no). The top half of each circle displays the interpretation of each psychometric characteristic (e.g., negligible/low/moderate/high/mixed; poor/good; or yes/no for task development to indicate whether this process was documented). The lower side of each circle displays the task types (e.g., stop-signal task, SST; Stroop; continuous performance task, CPT; Flanker; or Other, if the task could not be categorised into any of the previously mentioned task types). For example, in Fig. 4A, most of the studies using a continuous performance task (CPT) have low or negligible convergent validity, with fewer reporting moderate convergent validity.

For this review, we were interested to assess the psychometric characteristics of the gamified tasks. A summary of the psychometric characteristics of the gamified tasks is shown in Fig. 4.

### 3.2 Convergent validity

Most studies measured convergent validity (15/23 65 %), which refers to significant and substantial correlations between different instruments which aim to assess the same construct (Campbell and Fiske, 1959). To interpret Pearson’s correlations, coefficients from 0 to.30 (or 0 to −.30) were categorised as ‘negligible’, between.30 to 0.50 (or −.30 to −.50) as ‘low’, between.50 to.70 (or −.50 to −.70) as ‘moderate’, between.70 to.90 (or −.70 to −.90) as ‘high’ and between.90 to 1.00 (or −.90 to −1.00) as ‘very high’ (Mukaka, 2012).

Performance on gamified tasks in twelve studies (12/15, 80 %) correlated with equivalent, non-gamified tasks and self-report measures or did not significantly differ from equivalent standardised tasks or self-report measures (Brkic et al., 2022, Chicchi Giglioli et al., 2018, Chicchi Giglioli et al., 2021, Crepaldi et al., 2020a, Crepaldi et al., 2020b, Friehs et al., 2020, Gallagher et al., 2023, Johann and Karbach, 2018, Lumsden et al., 2017, Tong et al., 2021, Vladisauskas et al., 2024, Wang et al., 2023). Specifically, eight studies (8/15, 53 %) reported low or negligeable convergent validity. Brkic et al. (2022) reported that Go accuracy negatively correlated with inattention (r=-.43, p<.02) and executive functions (r=-.46, p<.008). Crepaldi et al. (2020a) reported that the total number of errors on the computer-based task correlated with the number of errors in the gamified task (rho=.44, p=0.04). Crepaldi et al. (2020b) reported that anticipation errors on the computer task correlated with those on the gamified task (rho=.0.37, p<.05) and with Stroop errors (rho=.43, p<.01). They also reported that omission errors on the computer task correlated with Stroop errors (rho=.36, p<.05). Johann and Karbach (2018) found significant correlations between metrics on the standardised Go/No-Go task and the gamified Go/No-Go task: commission errors (r=.38, p<.05), omission errors (r=.38, p<.05), RT Go (r=.70, <.001). They also report significant correlations between the standardised and the gamified Flanker task: ACC incongruent (r=.36, p<.05), RT congruent (r=0.44, p<.01, RT incongruent (r=.48, p<.01). Wang et al. (2023) reported that performance on the gamified and standard task correlated (r=.40, p<.001). Gallagher et al. (2023) reported a statistically significant correlation between the stop-signal reaction time and an impulsive/hyperactivity subscale (r=.36, p=.037). Chicchi Giglioli et al. (2021) found a significant correlation between latency in the gamified task, the non-planning subscale of the Barrett Impulsiveness Scale (r=-.40, p<.01), the standardised Dot Probe task (r=-.38, p<.01) and the standardised Stroop task (r=.32, p<.05), as well as a significant correlation between latency time on the gamified Go/No-Go task and latency time on the standardised Go/No-Go task (r=.31, p<.05). Finally, Vladisauskas et al. (2024) reported a negligeable correlation between accuracy on the Stroop and Flanker tasks (r=.29, p<.05), and accuracy on the Stroop task and RT on the Flanker task (r=.19, p<.05.). Nonetheless, they also reported a low correlation between RT on the Stroop and Flanker tasks (r=.44, p<.05). Only one study reported a moderate correlation (1/15, 7 %) between standard and gamified task performance (r=.69, p<.01) (Tong et al., 2021), and one study (1/14, 7 %) reported mixed findings, i.e., a negligible correlation between latency for Go trials on the gamified CPT task task and a standard CPT task (r=.129, p<.05), as well as a low correlation between latency time on their gamified Stroop task and a standardised Stroop task (r=.424, p<.01) (Chicchi Giglioli et al., 2018). Lastly, two studies found that task performance on their gamified SST tasks did not significantly differ from performance on a standardised SST (Friehs et al., 2020, Lumsden et al., 2017). Three studies (3/15, 20 %) reported poor convergent validity – one (Axelsson et al., 2016) reported that participants were able to better inhibit distractions in the gamified task compared with the standardised task (antisaccade task), one (Schroeder et al., 2021) found longer reaction times in a gamified stop-signal task compared with the non-gamified condition, and one study (Delgado et al., 2016) did not find any significant correlations between the two inhibitory control tasks and relevant Wechsler Intelligence Scale for Children (WISC-III) subscales.

From the studies that assessed convergent validity, 53 % (8/15) compared the gamified tasks with an equivalent standardised task. Most studies (87.5 %, 7/8) reported significant correlations between outcomes in the gamified and standardised tasks (CPT and Stroop: Chicchi Giglioli et al., 2018, Chicchi Giglioli et al., 2021; SST: Friehs et al., 2020; CPT: Johann and Karbach, 2018; SST: Lumsden et al., 2017; CPT: Tong et al., 2021; SST: Wang et al., 2023). Only one study reported significantly longer SSRTs and longer RTs in Go trials of a gamified version of the SST, and this was especially pronounced in overweight participants (Schroeder et al., 2021).

### 3.3 Discriminant validity

Discriminant validity of the tasks was also assessed in 7/23 (30 %) tasks. This measure aims to check that two instruments that measure a similar, but distinct trait are not correlated too strongly. To establish the discriminant validity of a measure, it is not sufficient to have low or near zero correlation coefficients, but also to make sure that correlations with scores on discriminant measures are noticeably lower than correlations with scores on convergent measures (Hubley, 2014).

Three tasks reporting discriminant validity indicated good levels (3/7, 43 %), meaning either that the task was successful in differentiating between cases (ADHD) and controls (Peijnenborgh et al., 2016), or that task performance was not significantly correlated with tasks measuring other constructs (Tong et al., 2021, Wang et al., 2023). Three studies had mixed results (3/7, 43 %). Chicchi Giglioli et al. (2021) reported a negligeable correlation between correct answers on the gamified Stroop task and correct answers on the Trail Making Test (r=.298, p<.05) and latency time on the gamified Stroop task had a low correlation with the Tower of London (r=.422, p<.01). However, the gamified CPT task had good discriminant validity, with no correlations with tasks measuring other constructs. Similarly, Delgado et al. (2016) used two tasks. The behavioural inhibition task had good discriminant validity while the CPT task was moderately correlated with the digit span subscale on the WISC-III (r=.55, p<.01). Finally, Vladisauskas et al. (2024) used two tasks. They reported significant correlations between accuracy on the Stroop task and planning (Tower of London) (r=.15, p<.05), and accuracy on the Flanker task and planning (r=.42, p<.05). Significant correlations between RT on the Stroop task and the working memory score (r=.13, p<.05), and between RT on the Stroop task and the working memory task (r=.31, p<.05), as well as between accuracy on the Stroop task and RT on the working memory task (r=-.16, p<.05) and between RT on the Flanker task and RT on the working memory task (r=.17, p<.05). One study (Heemskerk and Roebers, 2023) reported poor discriminant validity (1/7, 14 %), with significant correlations between RT on the Stroop task and a shifting task (r=.66, p<.001) and between Stroop accuracy and shifting accuracy (r=.18, p<.05).

### 3.4 Internal consistency

Internal consistency was also assessed in a small number of studies (3/23, 13 %). The internal consistency of a task can be assessed using the split-half approach or Cronbach’s alpha. The split-half approach involves the sub-division of the task data into two datasets (e.g., odd and even trials) such that the measures of interest can be computed separately for each of the two datasets. To obtain a measure of internal consistency, the measures from the odd and even datasets are correlated using a Person correlation corrected with the Spearman-Brown formula (r_sb_). Following conventions in the field, internal consistency coefficients below 0.5 were categorised as ‘low’, coefficients between 0.5 and 0.7 as ‘moderate’ and coefficients above 0.7 as ‘good’. On the other hand, Cronbach’s alpha coefficients between 0.70 and 0.95 are typically considered acceptable or high (Tavakol and Dennick, 2011), though values higher than 0.90 might signal item redundancy (Streiner, 2003).

Irrespective of the measure used, the three studies reporting on this indicated good internal consistency with high split-half reliability for gamified CPT, Flanker and Stroop tasks, r_sb_=.78–.99 (Johann and Karbach, 2018), high split-half reliability for a gamified Stop Signal task, r_sb_ =.83 (Wang et al., 2023) and high internal consistency for a behavioural inhibition task and a continuous performance task, α =.83–.98 (Delgado et al., 2016).

### 3.5 Test-retest reliability

Test-retest reliability is commonly estimated using two approaches – the interclass correlation (ICC) and Pearson correlations between the measures of interest at different timepoints. The ICC represents the ratio of variability between participants to the total variability, including participant and error variability. Although the two approaches often yield similar conclusions, the ICC differs from Pearson correlations in that it can estimate the agreement between measures while also capturing differences in the means of the compared scores (e.g., which can arise due to training effects over time) (Koo and Li, 2016). In line with conventions, ICC scores below 0.5 were categorised as ‘low’, scores between 0.5 and 0.75 as ‘moderate’, and above 0.75 as ‘good’ (Koo and Li, 2016).

Although three studies collected longitudinal measurements (Brkic et al., 2022, Lumsden et al., 2017, Smittenaar et al., 2015) and one administered the task twice on the same day (Friehs et al., 2021), only one measured test-retest reliability (1/3, 33 %), reporting a moderate interclass correlation for the SSRT (Stop Signal task, ICC =.60 for 64 trials) (Smittenaar et al., 2015).

#### 3.5.1 User experience in gamified tasks

Due to the novel nature of the tasks, user experience was also assessed. Under this category we report information on feasibility, acceptability and task development, where such information was available. Feasibility refers to whether “something can be done, should we proceed with it and if so, how” (Eldridge, Lancaster, et al., 2016). Some of the common indicators for assessing feasibility are completion rates, inconvenience and reasons for non-completion (Eldridge, Chan, et al., 2016). Acceptability refers to whether participants consider an intervention or a task appropriate, based on anticipated or experienced responses to the task (Sekhon et al., 2017). Specifically, it has been proposed that acceptability is a multicomponent construct, consisting of seven sub-components, namely affective attitude, burden, perceived effectiveness, ethicality, intervention coherence, opportunity costs and self-efficacy (Sekhon et al., 2017).

Ten studies assessed feasibility (10/23, 44 %). The preferred indicators of feasibility were completion rates. For ease of interpretation despite a lack of guidelines on evaluating completion rates, we consider a completion rate <50 % as ‘low’, between 50 % and 70 % as ‘moderate’ and >70 % as ‘high’. Based on these categorisations, three studies (3/10, 30 %) reported moderate (55–69 %) (Axelsson et al., 2016, Brkic et al., 2022, Smittenaar et al., 2015) and seven (7/10, 70 %) reported high completion rates (75–100 %) (Bhavnani et al., 2019, Chicchi Giglioli et al., 2018, Crepaldi et al., 2020b, Friehs et al., 2021, Friehs et al., 2022, Heemskerk and Roebers, 2023, Vladisauskas et al., 2024).

Seven studies assessed acceptability (7/23, 30 %). There was high heterogeneity in the measures used to assess acceptability, ranging from user experience interviews to the User Experience Questionnaire, the Revised Gameplay Questionnaire, the Enjoyment and Engagement questionnaire, the Intrinsic motivation inventory or the Flow State scale. Four studies (4/7, 57 %) reported high acceptability, referring to high levels of task acceptance, enjoyment, intrinsic motivation and experiences of flow (Bhavnani et al., 2019, Crepaldi et al., 2020b, Friehs et al., 2020, Wang et al., 2023); one study (1/7, 14 %) was found to be only partly acceptable, meaning that participants showed higher interest and perceived competence on the gamified task, but there were no differences between the gamified and standard tasks on effort, autonomy and relatedness (Johann and Karbach, 2018); lastly, two studies (2/7, 29 %) reported low acceptability for the gamified task, quantified as less enjoyment with the gamified task (Lumsden et al., 2017) and no differences in attractiveness, perspicuity, dependability, stimulation or novelty between the standard and the gamified tasks (Schroeder et al., 2021). Finally, twelve studies described how the tasks were developed (13/23, 57 %).

#### 3.5.2 Quality appraisal of gamified tasks

The included studies were assessed using the Appraisal tool for Cross-Sectional Studies (AXIS). The most common reasons on which studies were marked down were sample size justification (only 6/23, 29 % provided a power calculation) and the description of non-responders (only 11/23, 48 % categorised non-responders).

### 3.6 Virtual reality inhibition tasks

#### 3.6.1 Setting

The studies were conducted in different settings, including the United States (N = 10), Canada (N = 4), Spain (N = 8), Germany (N = 2), Romania (N = 2), UK (N = 2), Taiwan (N = 1), and South Korea (N = 1).

#### 3.6.2 Participant characteristics

Sample sizes ranged from 20 to 1469 participants (median = 78, IQR = 49.25), and included 5034 participants. Overall, 4600 typically developing (median = 52.5, IQR = 55), 355 individuals with ADHD (M = 44.4, SD = 27.41), 25 individuals with sports concussions, 24 individuals with TBI and 30 individuals with orthopedic injuries were included. Ages ranged from 6 to 90 years (see Fig. 7 for the mean age distribution). Female participants comprised 46 % of the overall sample. Regarding developmental stages, one study included preschool children (Bailey, 2021), fifteen studies included elementary school children and adolescents (Adams et al., 2009, Areces et al., 2018, Chen et al., 2023, Fernández-Martín et al., 2024, Hong et al., 2020, Iriarte et al., 2016, Lalonde et al., 2013, Mangalmurti et al., 2020, Muhlberger et al., 2020, Neguț et al., 2016, Nolin et al., 2012, Nolin et al., 2016, Parsons et al., 2007a, Rodrigues, 2016, Shen et al., 2022), one study included adolescents (Camacho-Conde and Climent, 2022), thirteen studies included adults (Alexander et al., 2024, Areces et al., 2019, Chicchi Giglioli et al., 2021, Climent et al., 2021, Donahue and Shrestha, 2019, Henry et al., 2012a, Parsons et al., 2013, Parsons and Barnett, 2019, Parsons and Barnett, 2018, Parsons and Carlew, 2016, Voinescu et al., 2023, Voinescu et al., 2023, Wiebe et al., 2023) and one study included older adults (Parsons and Barnett, 2019). Some studies did not report the mean age of participants (Chen et al., 2023, Nolin et al., 2016), the exact age ranges (Bailey et al., 2019, Chen et al., 2023, Fernández-Martín et al., 2024, Hong et al., 2022, Muhlberger et al., 2020, Nolin et al., 2012, Parsons and Barnett, 2019, Rodríguez et al., 2018) nor the gender split of the sample (Chen et al., 2023) and these are not included in the calculations.

#### 3.6.3 Types of tasks

**Fig. 5:**
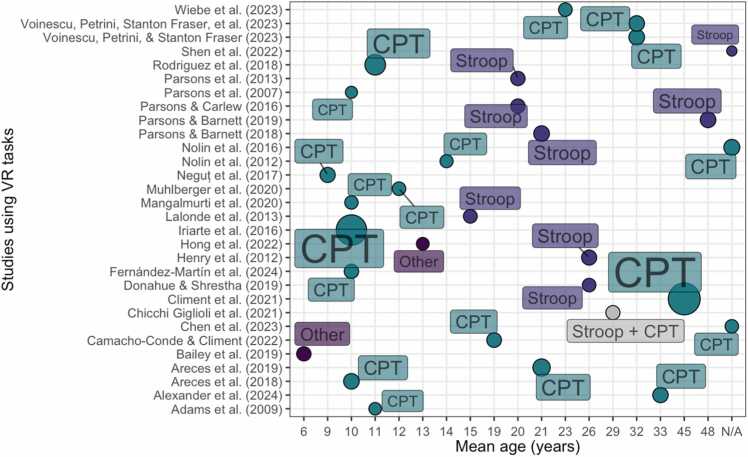
Tasks used in the virtual reality category and mean age (years, rounded) of the samples tested. The size of the circles provides an approximate estimation of sample size (i.e., a larger circumference indicates a larger sample size).

Tasks are summarised in Fig. 5. Overall, nineteen of the included studies employed continuous performance tasks and eight studies used Stroop tasks, with one study employing a rapid visual information processing task.

#### 3.6.4 Psychometric properties of the virtual reality tasks

**Fig. 6:**
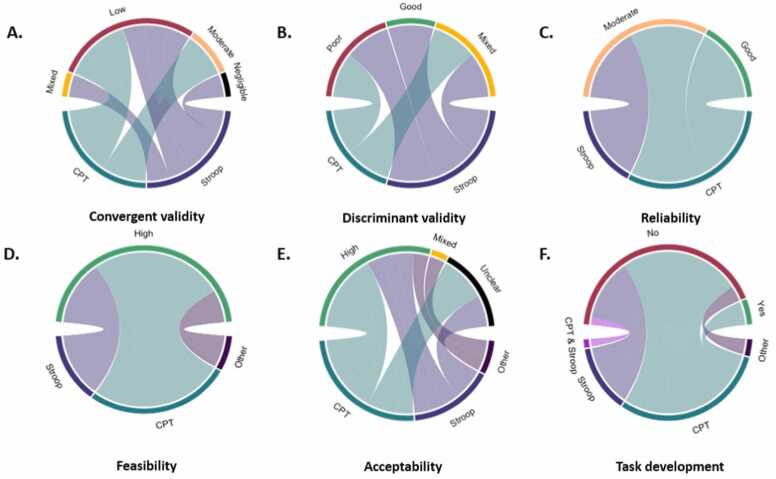
The psychometric characteristics of the various task types in the virtual reality category: (A) convergent validity, (B) discriminant validity, (C) reliability, (D) feasibility, (E) acceptability, (F) provided information on task development (yes/no). The top half of each circle displays the interpretation of each psychometric characteristic (e.g., negligible/low/moderate/high/mixed; poor/good; or yes/no for task development to indicate whether this process was documented). The lower side of each circle displays the task types (e.g., stop-signal task, SST; Stroop; continuous performance task, CPT; Flanker; or Other, if the task could not be categorised into any of the previously mentioned task types).

A summary of the psychometric characteristics of the virtual reality tasks is shown in Fig. 6.

### 3.7 Convergent validity

Approximately half of the studies assessed convergent validity (14/30, 47 %). One study (Bailey et al., 2019) had poor convergent validity (1/14, 7 %), as they reported a significant difference between the standard task (TV) and the VR task, with children demonstrating better inhibition in the TV task (t(42) = 2.99, p<.01, R2 = 0.26). Two studies reported mixed results (2/14, 14 %). Shen et al. (2022) reported mixed findings regarding convergent validity, with a low correlation between the VR task and the NIH toolbox (r=.49, p<.01) but no significant correlation between the VR task and parent-reported BRIEF scores. Neguț et al. (2016) reported mixed findings regarding convergent validity, with no significant differences between the VR and the standard task on omissions, commissions, and total correct responses for ADHD group, but slower RT in VR (p<.01, d=2.05). The same pattern of results held true for HCs, with significant differences between the two tasks in RT (slower in VR) (p<.01, d=2.04). The rest of the studies reported good convergent validity between the VR tasks and standardised tasks or questionnaires (11/14, 79 %). Muhlberger et al. (2020) found negligeable to moderate correlations between the CPT VR task and experimenter and parent reports, including omission errors in the VR task and attention measured by the experimenter (r=.029, p<.05) in the ADHD group, head movements in the VR task and hyperactivity measured by the experimenter (r=.51, p<.001) and parents (r=.32, p<.05) in the ADHD group, and between head movements in VR and hyperactivity measured by the experimenter (r=.44, p<.01). Nolin et al. (2016) reported low and moderate correlations between the CPT VR task and a computer CPT, including significant correlations between correct responses (r=.63, p<.001), commissions (r=.50, p<.001) and RT (r=.82, p<.001). Parsons and Barnett (2018) reported negligible correlations between accuracy scores in the VR Stroop task and scores on a standard Stroop task (r =.29, p<.01) and scores on the D-KEFS scale (r=.22, p<.05), and a negligible correlation between RT on the VR task and the D-KEFS (r=.21, p<.05). Donahue and Shrestha (2019) reported low and moderate correlations between the colour-word (CW) interference in the VR task and the Stroop word (r=.44, p<.01), Stroop Colour (r=.44, p<.01), Stroop-CW (r=.61, p<.001) and Stroop-Interference (r=.49, p<.01) on a standardised Stroop task.

There were further negligible and low correlations between VR-Colour and Stroop Word (r=.36, p<.05), Stroop-Colour (r=.42, p<.05) and Stroop-CW (r=.44, p<.01) on the standardised Stroop task, as well as between VR-Word and Stroop-CW (r=.40, p<.05). Voinescu, Petrini, Stanton Fraser, et al. (2023) reported low and negligible correlations between VR RT for correct responses and CPT RT for correct responses (r=.38, p<.01) and CPT commission errors (r=-.36, p<.01); VR commission errors and CPT RT for correct responses (r=.29, p<.01) and CPT commission errors (r=.49, p<.01); and VR omission errors and CPT RT for correct responses (r=.47, p<.01) and CPT omission errors (r=.48, p<.01). Adams et al. (2009) reported a moderate correlation between correct responses in VR and on the standard CPT task (rs(33)=.64, p<.001). Similar results were reported by Parsons et al. (2007), with a moderate correlation between commission errors in VR and errors (r=.51) and RT hits on the Conner's CPT (r=.75). Parsons, Courtney and Dawson (2013) reported low correlations between interference accuracy scores in VR and a computer-based Stroop (r=.38, p<.01) and the paper and pencil D-KEFS (r=.45, p<.01). Finally, reported low correlations between correct answers in VR and correct answers on a standard Go/No-Go task (r=.48, p<.01) as well as latency time on AT3 sig correlated with latency time on a Dot Probe task. Henry et al. (2012b) reported low correlations between correct responses (r = −0.46, p = 0.004), RT on correct responses (r = 0.38, p = 0.02), commission (r= 0.48, p = 0.003) and omissions (r = 0.47, p = 0.003) on the VR Stroop task (condition 2 - congruent and incongruent coloured words) and the same measures on a standard Stroop task. Parsons et al. (2007) reported moderate and high correlations between commission errors in the VR task and errors (r=.51) and RT hits on the Conner's CPT (r=.75), as well as moderate correlations between omission (r=.51) and commission errors in the VR task (r=.59) and score on the SWAN. Chicchi Giglioli et al. (2021) found that correct answers on AT3 significantly correlated with correct answers on a standard Go/No-Go task (r=.48, p<.01) and latency time on AT3 significantly correlated with latency time on Dot Probe task (r =.36, p<.05). They also found that correct answers and latency time on the AT4 significantly correlated with correct answers (r=.72, p<.01) and latency time (r=.31, p<.05) on a standardised Stroop task. Correct answers on the AT4 also correlated with correct answers on the standardised Go/No-Go task (r=.35, p<.05) and latency time on the AT4 correlated with latency time on the Dot Probe task (r=.35, p<.05). Voinescu, Petrini, Stanton Fraser, et al. (2023) reported mixed findings. Most correlations between the VR CPT and the standard neuropsychological tests were weak or non-significant. However, they reported medium correlations between TMT-A and omission errors in VR (r=.53, p<.01) and TMT-B and omission errors in VR (r=.63, p<.01) and a low correlation between spatial working memory span and omissions (r=-.36, p<.01). Parsons and Carlew (2016) found no significant differences between groups for any Stroop modality (F(1,15) = 2.50, p=.134).

From the studies that assessed convergent validity, 71 % (10/14) compared the gamified tasks with an equivalent standardised task. Most studies (90 %, 9/10) reported significant correlations between the VR tasks and a standardised equivalent (CPT: Adams et al., 2009; CPT and Stroop: Chicchi Giglioli et al., 2021; Stroop: Donahue and Shrestha, 2019; Stroop: Henry et al., 2012a; Stroop: Parsons et al., 2007b, Parsons et al., 2013; Stroop: Parsons and Barnett, 2018; Stroop: Parsons and Carlew, 2016; CPT: Voinescu, Petrini, Stanton Fraser, et al., 2023). Only one study (10 %) using a Simon Says task reported a significant difference between the two tasks, with the conventional task eliciting better inhibitory control than the VR task (Bailey et al., 2019). From the studies that found the two tasks to be comparable, three studies (30 %) compared performance on standardised and VR tasks in typically developing and neurodivergent individuals. One study reported no significant difference between the standardised and the VR tasks in typically developing individuals but found that individuals with high functioning autism performed worse in VR (Parsons and Carlew, 2016). Two studies investigated ADHD and reported that individuals with ADHD performed worse than controls in the VR condition (Parsons et al., 2007b), though one only found a trend difference (Adams et al., 2009).

### 3.8 Discriminant validity

Only a few of the included studies assessed discriminant validity (5/30, 17 %), and most of them (4/5, 80 %) assessed mixed-domain constructs. Lalonde et al. (2013) reported poor discriminant validity, as higher numbers of violations on the planning subscale of the D-KEFS were associated with more commission errors in the VR task (beta =.52, SE=1.54, t=3.63, p =.001). Chicchi Giglioli et al. (2021) also reported low discriminant validity, as correct answers on AT3 significantly correlated with latency time (r=.31, p<.05) and with preservative responses (r=-.32, p<.05) on the Wisconsin Card Sorting Task, and with initial time on the Tower of London task (r=.30, p<.05). Two studies reported mixed results. Voinescu, Petrini, Stanton Fraser, et al. (2023) reported mixed findings, with most correlations between VR CPT measures and neuropsychological tests being weak or non-significant. However, they reported significant moderate correlations between TMT-A and omission errors in VR (r=.53, p<.01) and TMT-B and omission errors in VR (r=.63, p<.01) and a significant low association between spatial working memory span and omissions (r=-.36, p<.01). Similarly, Donahue and Shrestha (2019) found no associations between the VR Stroop task and ACS-focusing, but found low to moderate correlations between TMT-A and VR-Word (r=-.37, p<.05), VR-Colour (r=-.38, p<.05) and VR-CW (r=-.42, p<.05), between TMT-B and VR-Word (r=-.45, p<.01), VR-Colour (r=-.37, p<.05) and VR-CW (r=-.57, p<.001), as well as between ACS-Shifting and VR-CW (r=.37, p<.05). Parsons and Barnett (2018) reported good discriminant validity, with no significant correlations between interference in the VR task and scores on a learning (r=-0.04, p=0.73) and two delay free recall scales (r=-0.15, p=0.16, r=-0.03, p =0.82).

### 3.9 Test-retest reliability

Two studies measured test-retest reliability (2/30, 7 %). Nolin et al. (2016) reported moderate and low correlations for time 1 (T1) and time 2 (T2) measured one month apart between correct response (r=.61, p<.001), commission (r=.34, p<.05), right and left head movement (r=.49, p<.01), up and down head movement (.54, p<.001), tilt head movement (r=.46, p<.01). Shen et al. (2022) reported moderate test-retest reliability (ICC =.63) for T1 and T2 measured three weeks apart.

### 3.10 Internal consistency

Only one study assessed internal consistency (1/30, 3 %), and reported Cronbach's alpha =.72 (Rodríguez et al., 2018) indicating acceptable consistency.

#### 3.10.1 User experience

More than half of the included studies assessed acceptability (16/30, 53 %). The measures most used to assess acceptability in the virtual reality studies were the Simulation Sickness Questionnaire (9/16, 56 %) and the Presence Questionnaire (6/16, 38 %). More than half of the studies (9/16, 56 %) used more than one measure to assess acceptability. Nine studies reported high acceptability (9/16, 56 %), referring to high levels of task enjoyment, good sense of presence, adequate realism and few cybersickness symptoms (Bailey et al., 2019; Donahue and Shrestha, 2019; Henry et al., 2012b; Neguț et al., 2016; Nolin et al., 2012, Nolin et al., 2016; Shen et al., 2022; Voinescu, Petrini, and Stanton Fraser, 2023; Voinescu, Petrini, Stanton Fraser, et al., 2023). The most reported cybersickness symptoms were eye strain and fatigue. Four studies did not provide details on acceptability, although the authors declared that participants did not report any significant post-exposure VR sickness (Adams et al., 2009, Lalonde et al., 2013; Parsons et al., 2007; Parsons and Carlew, 2016). One study administered the Simulation Sickness Questionnaire but did not report results (Parsons et al., 2013). Lastly, in one study adolescent participants reported a good sense of presence but high cybersickness symptoms (Hong et al., 2022).

Six studies included information on feasibility (7/30, 23 %), reporting high compliance among their participants (63–100 % compliance) (Bailey, 2021, Fernández-Martín et al., 2024, Muhlberger et al., 2020, Neguț et al., 2016, Parsons and Barnett, 2019, Parsons and Barnett, 2018, Voinescu et al., 2023). Finally, only three studies provided details on task development (3/30, 10 %) (Alexander et al., 2024, Camacho-Conde and Climent, 2022, Wiebe et al., 2023).

#### 3.10.2 Quality appraisal

The included studies were assessed using the Appraisal tool for Cross-Sectional Studies (AXIS). The most common reasons on which studies were marked down were sample size justification (only 1/30, 10 % studies provided a power calculation) and the description of non-responders or excluded participants (10/30, 33 %).

### 3.11 Ecological momentary assessment

#### 3.11.1 Setting

Most of the included studies were conducted in the United States (N = 4), France (N = 3), and Israel (N = 3), with one study being conducted in the United Kingdom and one in Australia.

#### 3.11.2 Participant characteristics

Sample sizes ranged from 12 to 283 participants (M = 101.17, SD = 69). Overall, 224 healthy controls were included (M = 84.83, SD = 75.33). Ages ranged from 9 to 75 years (M= 31.64, SD = 10.56). Warren and Peltz (2019) included a sample of 7th graders but did not explicitly report their age. Regarding developmental stages, ten studies included adults, from youth to older adults (Ben-Dor Cohen et al., 2023, Bouvard et al., 2018, Chirokoff et al., 2024, Chirokoff et al., 2024, Dali et al., 2024, Nahum et al., 2023, Powell et al., 2017, Sobolev et al., 2021, Tseng et al., 2020, Yitzhak et al., 2023), and two studies included elementary school children and adolescents (Chaku et al., 2024, Warren and Pentz, 2019). In total, the included studies included 752 female participants (62 %).

#### 3.11.3 Task characteristics

**Fig. 7:**
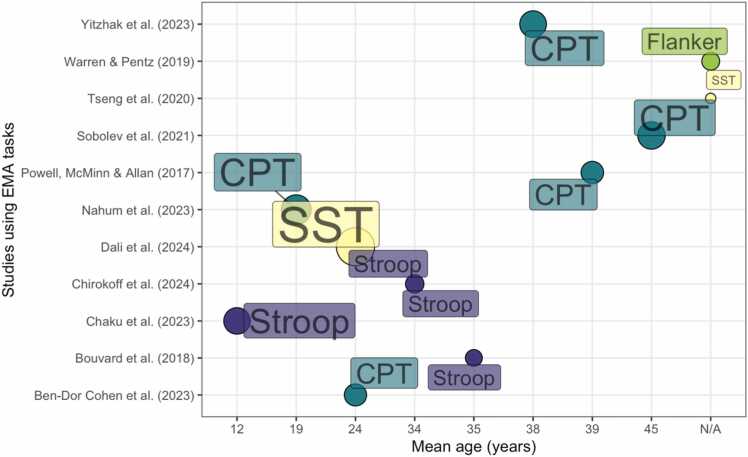
Tasks used in the EMA category and mean age (years, rounded) of the samples tested.

Tasks are summarised in Fig. 7. The tasks were delivered on wrist-worn devices (N=1), smartphones (N=10), computer (N=1), and a combination of smartphones and computers (N=1). Five studies used an EMA version of a CPT (Ben-Dor Cohen et al., 2023, Nahum et al., 2023, Powell et al., 2017, Sobolev et al., 2021, Yitzhak et al., 2023), one study used a Flanker task (Warren and Pentz, 2019), four studies used Stroop tasks (Bouvard et al., 2018, Chaku et al., 2024, Chirokoff et al., 2024, Chirokoff et al., 2024) and two studies used a SST task (Dali et al., 2024, Tseng et al., 2020).

The length of the studies varied between 3 and 28 days (median = 14 days, IQR = 16.75). Two of the included studies used continuous sampling, once every hour (Powell et al., 2017, Tseng et al., 2020) and the other two used random sampling (Sobolev et al., 2021, Warren and Pentz, 2019). Of these using random sampling, one prompted participants randomly in the morning and evening (Sobolev et al., 2021), and one sent two prompts between 3 and 10 pm (Warren and Pentz, 2019). Most studies incentivised participants for their participation, with two offering flat payments (Powell et al., 2017, Sobolev et al., 2021) and one paying participants per prompt (Tseng et al., 2020). Compliance varied between 57 % and 97.25 %. Two studies reported their allowed response delay, which varied between 20 minutes (Powell et al., 2017) and 1 hour (Tseng et al., 2020).

Most of the included studies did not report task duration; however, one study reported the duration of the entire EMA battery, which was 5.24 minutes (SD = 2.38) (Warren and Pentz, 2019). Only one study included a training video for their participants (Sobolev et al., 2021), and one other study included practice trials for the EMA task (Tseng et al., 2020).

Most studies in this category focused on understanding the relationship between inhibitory control and health behaviours (2/4; snacking behaviour in adults, 1/2; and sedentary behaviour in adolescents, 1/2). Two studies focused on monitoring inhibitory control with the view that it could aid the management of psychiatric conditions (2/4).

The size of the circles provides an approximate estimation of sample size (i.e., a larger circumference indicates a larger sample size).

#### 3.11.4 Psychometric properties of the ecological momentary tasks

**Fig. 8:**
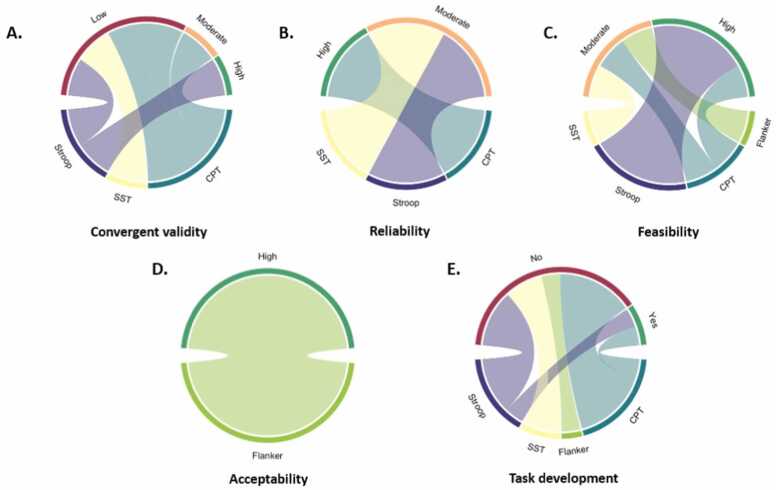
The psychometric characteristics of the various task types in the ecological momentary assessment category: (A) convergent validity, (B) reliability, (C) feasibility, (D) acceptability, (E) provided information on task development (yes/no). The top half of each circle displays the interpretation of each psychometric characteristic (e.g., negligible/low/moderate/high/mixed; poor/good; or yes/no for task development to indicate whether this process was documented). The lower side of each circle displays the task types (e.g., stop-signal task, SST; Stroop; continuous performance task, CPT; Flanker; or Other, if the task could not be categorised into any of the previously mentioned task types).

A summary of the psychometric characteristics of the ecological momentary tasks is shown in Fig. 8.

### 3.12 Convergent validity

Six studies (6/12, 50 %) assessed convergent validity (Ben-Dor Cohen et al., 2023, Bouvard et al., 2018, Chaku et al., 2024, Sobolev et al., 2021, Tseng et al., 2020, Yitzhak et al., 2023). Sobolev et al. (2021) reported low convergent validity (r =.47, p<.001) between reaction time on the standardised task and reaction time on the EMA task, and Tseng et al. (2020) reported that the individual SSRT on the EMA task and self-reported inhibitory control at baseline were inversely correlated (b = 1.04, p<.001), signaling low convergent validity. Similarly, Ben-Dor Cohen et al. (2023) reported a low correlation (r=.46) between the EMA CPT and baseline CPT, and Chaku et al. (2023) found low correlations between the EMA Stroop and attentional control at baseline for each participant (r=.20, range:.01–.37). Finally, Yitzhak et al. (2023) reported a moderate correlation between the full-length CPT and the EMA CPT (r=.62) and Bouvard et al. (2018) found a high correlation between the EMA Stroop and the standard Stroop task (colour-word Stroop: r=.90; letter-word Stroop: r=.68).

### 3.13 Test-retest reliability

Three studies (3/12, 25 %) assessed test-retest reliability. Sobolev et al. (2021) reported high correlations between reaction times on the EMA task between baseline and morning (r = 0.88, p<.001), evening (r = 0.86, p<.001) and the end of the study (day 21) (r = 0.79, p<.001). Chaku et al. (2023) reported a moderate ICC for the inhibitory control score on the Stroop task across the 100 days of the study (ICC=.53) and a moderate ICC for the last 93 days of the study (excluding the first 7 days) (ICC=.56). Finally, Dali et al. (2024) reported a moderate ICC for SSRT between sessions 1 and 8 (ICC=.53).

#### 3.13.1 User experience

One study provided information on acceptability (1/12, 8 %) (Warren and Pentz, 2019). The study was found to be acceptable by participants, who reported the study activities as enjoyable and not burdensome. All studies provided information on feasibility, with participants completing between 19 % and 97.25 % of the scheduled prompts. Specifically, one study reported low feasibility (Tseng et al., 2020), three reported moderate feasibility (Dali et al., 2024, Nahum et al., 2023, Warren and Pentz, 2019) and seven reported high feasibility (Ben-Dor Cohen et al., 2023, Bouvard et al., 2018, Chaku et al., 2024, Chirokoff et al., 2024, Powell et al., 2017, Sobolev et al., 2021, Yitzhak et al., 2023). Finally, only two studies provided information on task development (Sobolev et al., 2021) (2/12, 17 %).

#### 3.13.2 Quality appraisal

The included studies were assessed using an EMA quality appraisal tool developed by Liao et al. (2020) and adapted by Kwasnicka et al. (2021). Overall, studies received a ‘Strong’ rating (12/12, 100 %) for the rationale provided for using an EMA design, ‘Strong’ rating (2/12, 17 %) for providing a power calculation, ‘Strong’ rating (7/12, 58 %) for adhering to the EMA protocol and a ‘Strong’ rating (3/12, 25 %) for the treatment of missing information.

## 4 Discussion

This systematic review summarises naturalistic studies using gamification, virtual reality and ecological momentary assessments to measure inhibitory control across development. We identified 64 studies that investigated inhibitory control either in the real-world, or by recreating the real-world in the laboratory. Continuous performance tasks were the most used tasks across all categories, followed by stop-signal tasks in the gamified category and Stroop tasks in the virtual reality and EMA categories. Negligible to moderate correlations were reported between naturalistic and equivalent standardised tasks or self-report measures in the gamified (r=.13–.69), virtual reality (r=.03–.82) and EMA (r=.20–.90) categories. However, a considerable proportion of studies in the gamified (50 %) and virtual reality (80 %) categories used tasks that appeared to measure mixed-domain constructs. Test-retest reliability varied from low to high across categories. Specifically, one study in the gamified category reported a high interclass correlation coefficient (ICC=.60), two studies in the virtual reality category reported low to moderate correlations (r=.34–.61) and a high ICC (.63). In the EMA category, one study reported high correlations between baseline and repeated assessments up until 21 days (r=.79–.88), and two reported moderate intraclass correlation coefficients between baseline and repeated assessments up to 100 days (ICC =.53), and between baseline and the last session (up to 24 days) (ICC =.53). Despite high heterogeneity in the types of tasks and psychometric details reported, most tasks were highly acceptable (67 % of the gamified tasks, 64 % of the virtual reality tasks, and one EMA task), referring to high enjoyment of the naturalistic tasks. Feasibility was high and frequently reported in the gamified category, with 70 % of studies reporting completion rates between 75 % and 100 %. In virtual reality, only 23 % studies reported details on feasibility, although these that did report had high completion rates (63–100 %). The EMA task prompts were completed in variable proportions, ranging from 19 % to 97 %. Overall, naturalistic and standardised tasks were generally comparable in terms of performance and participants’ enjoyment either did not differ or was enhanced when completing the naturalistic versions.

In typical experimental paradigms used in cognitive sciences, participants are required to complete computerised tasks that elicit the construct of interest (e.g., inhibitory control) using repeated, decontextualised stimuli that often do not resemble a response inhibition-related activity that the participants would ordinarily encounter in their everyday lives. This practice is likely based on several assumptions, including that real-world tasks introduce noise and confounding factors (Nastase et al., 2020) and that they are not psychometrically sound (Burgess et al., 2006). However, the current review suggests that most naturalistic tasks have acceptable psychometric properties, meaning that they seem to measure the construct they are aiming to measure. Where reported, both researchers and participants were generally enthusiastic about the naturalistic assessments, as shown by broadly high completion rates across all categories and high levels of participant engagement and motivation. These findings are similar to those of a review investigating gamification in cognitive assessment and training, which reported intertask correlations of r=.45–.60 in studies measuring broad cognitive function (Lumsden et al., 2016) as well as high participant engagement. Indeed, it is important to acknowledge that some tasks, especially these in the virtual reality category, correlated with measures of other cognitive domains, suggesting that these measures were, in fact, mixed-domain measures rather than pure inhibitory control measures. However, these results are based on a low number of studies reporting information on discriminant validity (5/27 studies in the VR, 5/21 in the gamified, and 0/4 in the EMA categories).

It is also notable that most of the included studies were conducted in the United States, with a substantial proportion of participants identifying as White ethnicity. This aligns with the findings of a systematic review and meta-analysis of ecological momentary assessment studies measuring health behaviours in context (Perski et al., 2022). It is also in line with other research reporting on the overreliance of psychological science on so-called WEIRD populations (Western, Educated, Industrialised, Rich and Democratic), although Western industrialised countries represent only 12 % of the world’s population (Arnett, 2008). Nonetheless, the included studies reported a relatively equal gender split and half of the studies sampled their participants from the general population. Most of the included studies delivered the tasks via technological tools, such as computers, tablets and smartphones. Tasks were mostly delivered via computers in the gamified category, smartphones in the EMA category and head-mounted displays in the VR category.

Based on the increase in published studies using gamified, virtual reality and EMA inhibitory control tasks in recent years, it is not unreasonable to assume that the prominence of non-naturalistic tasks in the cognitive science literature until recently could be explained, at least partly, by the high costs and reduced access, or unavailability of certain technologies required to access, develop and deploy naturalistic paradigms (Gibbons, 2017). For example, Hodgson et al. (2015) reported that the cost of comparable VR hardware from 2006 to 2014 decreased from 45,000 USD to 1300 USD, and a recent review of VR applications in higher education highlighted the increased accessibility of VR head-mounted displays to the general population was made possible by the reduced costs of headsets (approximately 400 USD) (Radianti et al., 2020). Similarly, smartphones have become increasingly affordable and prevalent worldwide, with more than one third of the global population owning one (GSMA intelligence, 2019). In the UK, it is estimated that approximately 84 % of the population has access to a smartphone, including more than 95 % of those aged 18–54 (Boyle and Barber, 2023). This means that smartphone-based ecological momentary assessments can be delivered more easily, and data can be collected more reliably (e.g., delayed responses can be automatically tracked in smartphone-based EMA compared with pen-and paper or older EMA devices such as palmtop devices). In this vein, whilst our original systematic search identified only four EMA studies, the highest proportion of new studies meeting the inclusion criteria for this review in the updated systematic search between February 2023 and September 2024 were EMA studies (8 out of the 13 newly identified studies). These data, along with a shift in the field towards the use of more naturalistic tasks (Allen et al., 2024, Hartley, 2022, Prasad, 2024, Vigliocco et al., 2023), indicates that more conclusive results could be drawn in the following years as more research using naturalistic cognitive tasks emerges.

### 4.1 Gamification of cognitive assessments

Traditional cognitive assessments of executive functioning have received several criticisms in recent years. For instance, it has been postulated that traditional cognitive assessments are insufficiently sensitive and low in ecological validity (e.g., individuals who are expected to display lower performance on cognitive assessments often do not differ from controls) (Burgess et al., 2006, Valladares-Rodríguez et al., 2016). They often require specialised training and are long and boring to complete for participants (Hu et al., 2022). This can be especially problematic since research indicates that a lack of participant motivation can negatively impact the quality of the collected data (DeRight and Jorgensen, 2015). Introducing game elements to executive functioning tasks has been proposed as one method of increasing intrinsic motivation, improving task engagement and, consequently, task performance (Dörrenbächer et al., 2014). Based on the studies included in the current review that assessed user experience, the gamified tasks were deemed acceptable and enjoyable by participants. Most studies provided information on task development, but task code was not commonly shared. Providing information on task development and sharing code is important to increase replicability and collaboration between research groups and across disciplines. Though most studies did not report the duration of the gamified assessments, the ones that did were generally under 10 minutes and one study using a task with a duration of only 4 minutes reported that it was the most popular among their participants (Smittenaar et al., 2015). Furthermore, some studies focused on the potential of using gamification to engage certain age groups more appropriately, such as older adults or young children. In fact, it has been suggested that gamified neuropsychological tasks are suitable for engaging children as young as 2 years old (Semmelmann et al., 2016) and that they provide a unique opportunity to create scalable, low-cost and cross-culturally valid tools to assess early childhood development (Mukherjee et al., 2020). These observations align with the findings of a recent systematic review, where the authors identified multiple reasons why researchers opt to use gamification for their cognitive testing. These included increasing participant motivation, increasing usability or intuitiveness for specific age groups (i.e., elderly, young children), increasing long-term engagement, increasing ecological validity, and increasing their suitability for specific conditions (e.g., ADHD) (Lumsden et al., 2017).

It is worth noting that most included studies had relatively small sample sizes, with the notable exception of a study that formed part of the Great Brain Experiment which has a sample of 22,098 participants (Smittenaar et al., 2015). The relatively small sample size is perhaps explained by the novelty of most tasks, and the fact that all but three studies collected data in person, which comes with time and access constraints. Nonetheless, games or gamified tasks show excellent potential for longitudinal assessment, as games are often revisited and can be used to assess change over time (Allen et al., 2024).

### 4.2 VR for cognitive assessments

Recent advancements in virtual reality enable researchers to bring more naturalistic environments into the laboratory. This is a unique advantage of virtual reality, because assessments can be developed to include elements or scenarios that individuals would naturally encounter in their everyday lives, whilst also maintaining experimental control (Neguț et al., 2016). Increasing the ecological validity of cognitive assessments is crucial, as performance on neuropsychological tests has been shown to only account for 4.6–21.4 % variance in daily functioning (Van der Elst et al., 2008). Approximately half of the studies included in this review assessed user experience (47 %). Nine of 16 studies reported high acceptability, referring to high levels of task enjoyment, good sense of presence, adequate realism and few cybersickness symptoms. The most reported cybersickness symptoms were eye strain and fatigue. Feasibility was assessed in a subset of the included studies, and all indicated moderate to high completion rates (63–100 %). Only a small proportion of the included studies (10 %) provided details on task development. Since the assessment of cognitive functioning using VR is a relatively new field, we strongly encourage researchers to share details on task development and technical decisions to help advance the field and foster collaboration.

### 4.3 EMA for cognitive assessments

Recent advancements in technology, increased affordability and prevalence of smartphones worldwide and the pandemic have led to a surge in mobile health (m-Health) research (Cao et al., 2021). In this review, we specifically focused on ecological momentary assessments. Complementary to VR, EMA enable researchers to bring the laboratory into people’s real lives and understand behaviour, cognition and affect more dynamically. Despite a surge in the number of EMA publications in recent years (Fig. 2), most EMA research focuses on affect and behaviour, leaving momentary cognition relatively unexplored. Cognitive ecological momentary assessments or ecological momentary cognitive tests (EMCT) refer to cognitive assessments that are brief, repeatable and can be self-administered via smartphones as participants go about their day-to-day lives. EMCT have evolved to address the limitations of traditional cognitive assessments, including reliance on one-time assessments that merely capture a snapshot of an individual’s cognitive functioning, reliance on retrospective assessment and lack of generalisability (e.g., for individuals who appear to perform well in controlled laboratory circumstances despite struggling in their everyday lives) (Singh et al., 2023). The current review identified twelve studies that assessed inhibitory control using EMA tasks - two focused on monitoring inhibition in relation to health behaviours such as snacking or sedentarism, four focused on monitoring in relation to psychiatric or neurodevelopmental conditions, three investigated the feasibility of using brief tasks to measure inhibitory control, and three focused on assessing the association between momentary fluctuations in inhibitory control and psychological constructs (e.g., resilience). Despite that a number of EMA studies focus on monitoring and identifying early warning signs of psychopathology (Helmich et al., 2022, Schreuder et al., 2020, Smit and Snippe, 2022), determining what constitutes an early warning sign can be challenging. Helmich et al. (2021) have proposed a conceptual checklist for designing studies on early warning signals in psychopathology, including considerations of how relevant transitions can be distinguished from normal variation or the ideal sampling interval for capturing fluctuations. Nonetheless, this can be particularly difficult in momentary cognition tasks, which have been less investigated compared with self-reported momentary affect. Daniëls et al. (2020) are among the few to have investigated momentary cognitive tasks to date and suggest that the level of difficulty of momentary cognitive tasks needs to be adjusted based on individual performance, to maintain engagement and flow. Moreover, they suggest that context and mood are assessed in parallel, to further disentangle their interactions with cognition.

Furthermore, it remains unclear which are the most suitable types of tasks that can be delivered using an experience sampling protocol. Given that tasks are completed repeatedly as the participants go about their day-to-day lives, it is important to consider practice effects, feasibility and user experience. In the current review, two studies used momentary CPT tasks. The choice of task was motivated by its ease of implementation (i.e., a single button press/tap) and the unpredictability of the task, which was deemed more appropriate for repeated assessments compared. Indeed, the Stroop task has been shown to lead to large practice effects if completed multiple times (Davidson et al., 2003). One other study included in the EMA category used a stop signal task. Like the CPT tasks, the stop signal task is used as a measure of response inhibition and has been shown to activate the fronto-striatal system (Hung et al., 2018). However, the stop signal task further involves an auditory or tactile stimulus that prompts the participant to inhibit their proponent response, which must be taken into consideration in an ambulatory protocol (e.g., introducing a visual stop signal). Finally, the last study included in the EMA category used a Flanker task. Flanker tasks have been used less frequently in the context of naturalistic assessments of inhibition, as evidenced by only one study employing a Flanker task in the EMA category and one other study in the gamification category. However, Flanker tasks have been used successfully in longitudinal research, showing good psychometric properties even when administered 2 years apart (Richardson et al., 2018). Nonetheless, it is important that its psychometric properties are investigated in the context of experience sampling research. In experience sampling, researchers are interested in fluctuations in the construct of interest (e.g., inhibitory control); therefore, it is important that reliability calculations are adapted to suit this design (e.g., see Dejonckheere et al., 2022).

Further considerations in EMA research include identifying and adequately managing careless responding. Careless responding can introduce non-random patterns in the data and lead to spurious correlations between variables (Huang et al., 2012). Some recommendations have been made, including keeping the length of the assessment short, examining within-person variance and response times (Eisele et al., 2022). While most research and recommendations to date have been formulated in relation to self-report EMA items, a recent study adapted three cognitive tasks for use on smartphones and showed that assessments between 60 and 90 seconds can provide reliable and valid measures of executive functioning (Perzl et al., 2023). Furthermore, it is important to consider that some of the advantages of momentary assessments can also act as disadvantages, especially for task assessing inhibition. For instance, participants may be distracted by notifications received on their smartphones or might abandon the task altogether in favour of competing interests.

Finally, experience sampling designs are intensive and burdensome for participants, such that incentive schemes and compliance rates are important considerations in EMA research. In the current review, the tasks were delivered for a median of 14 days, with two studies using random and two studies using continuous (hourly) sampling throughout the day. Two studies incentivised participants with a flat payment for completing at least 80 % of assessments, one study provided a payment for each completed prompt, and one did not provide any incentives. Not surprisingly, the study where participants were not incentivised had the lowest compliance rate (57 %). It is important that researchers interested in experience sampling designs choose an incentive scheme that is proportional with participants’ time to improve compliance rates.

## 5 Quality appraisal

The primary reason for which studies across all categories were marked down was the lack of an *a priori* power analysis to justify their sample size. To some extent, this can be explained by the pilot nature of some of the included studies. Pilot studies have the objective of estimating parameters for the main study, rather than proving the superiority of a treatment or procedure (Whitehead et al., 2014, Whitehead et al., 2016). Nonetheless, there are methods in place for calculating sample sizes for pilot studies when the standardised effect size for the main study is known, or guidance on the use of approximate rules if the effect is unknown (Whitehead et al., 2016). Tutorials for conducting power analyses in EMA research have also been published (Lafit et al., 2021), though their use appears limited at present, as has been noted in a recent review of EMA studies of health behaviours (Perski et al., 2022). Issues of low statistical power (Maxwell et al., 2015) have been linked to concerns about replicability. Increasing the methodological rigour and transparency of naturalistic research in cognitive sciences is imperative, especially considering the replicability, credibility and transparency concerns in recent years in the fields of psychology and neuroscience (Lewandowsky and Oberauer, 2020).

## 6 Implications and avenues for future research

Through increased engagement, naturalistic assessments are also promising for reducing attrition in longitudinal studies and have the potential to be customised for the specific needs of target populations (e.g., based on age or health condition) (e.g., Kalantari et al., 2022). Future research using naturalistic methods to study cognition would further benefit from integrating multiple technologies, for example gamification and virtual reality, or gamification and ecological momentary assessments (e.g., see Dietvorst et al., 2022 for a smartphone serious game for adolescents including ecological momentary assessments). Furthermore, deploying naturalistic assessments on a larger scale by making the assessments available remotely (e.g., by leveraging the existence of remote participant recruitment platforms such as Prolific or online experiment platforms such as Gorilla.sc) is a promising avenue for future research that warrants further exploration. Even when access to specialised hardware is necessary as is the case with VR, it has been shown that behavioural assessments can be feasibly delivered remotely (Clements et al., 2023, Huber and Gajos, 2020).

To aid data quality and collaboration, we encourage researchers to engage in Open Science practices, including pre-registering study protocols, data sharing (e.g., on the Open Science Framework) and sharing their tasks or questionnaires on suitable repositories (e.g., the ESM Item Repository for EMA items; https://www.esmitemrepositoryinfo.com/). Engaging in these practices can not only make research outputs less error-prone but can also make research more visible to researchers from the same or distinct disciplines, and to the general public (Armeni et al., 2021). Increasing collaboration is crucial since large sample sizes typically involve collaborative efforts from multiple investigators. This is especially valuable in the naturalistic study of cognition, as interdisciplinarity is central at the methodological level to ensure adequate development and implementations of naturalistic paradigms (Vigliocco et al., 2023).

Considering the extensive heterogeneity of the included studies and the tasks used to measure inhibitory control, the authors strongly encourage other researchers using or interested in adopting naturalistic methods to study cognition to make their paradigms and associated code available on repositories such as GitHub or to combine efforts to create a comprehensive database of naturalistic paradigms for executive functions. Here we note the existence of a repository for ecological momentary assessment measures (https://www.esmitemrepository.com/) and a naturalistic neuroimaging database (https://www.naturalistic-neuroimaging-database.org/), though they are not specific to executive functions.

For researchers interested in using EMA to study cognition, it might be useful to leverage advancements in sensor technology to identify contexts or locations that might influence inhibitory processes and health behaviours (e.g., Niemeijer et al., 2023). We recommend that EMA researchers take advantage of existing guidance and tools that can inform important study design decisions, such as sample size (Lafit et al., 2021) and sampling frequency (Eisele et al., 2022), or that advise on best practices for pre-registering studies using this methodology (Kirtley et al., 2021). Furthermore, future research might expand the application of momentary cognitive tasks to other age groups. In the current review, only one study investigated the use of a momentary inhibitory control task in non-adult populations. However, a recent meta-analysis found no significant effect of age on compliance in ecological momentary assessment studies, indicating that these designs could also be deployed in adolescents (Wrzus and Neubauer, 2023).

### 6.1 Strengths

First, a key strength of this review is the comprehensive summary of naturalistic inhibitory control assessments since records began and across three categories. To our knowledge, this is the first review summarising evidence on the use of naturalistic methods to assess inhibitory control across the lifespan. Second, we provide an overview of the psychological constructs assessed across the included studies, highlighting differences in focus across the categories and identifying potential gaps for future research. Thirdly, we assess the quality of the included studies using two quality appraisal tools, namely the widely used AXIS for cross-sectional studies and a quality appraisal tool specifically designed for EMA studies by Kwasnicka et al. (2021). Fourth, this review was conducted by a team of researchers working in related, but distinct and complementary branches of neuroscience and psychological sciences. Fifth, Open Science principles were followed throughout all the stages of the review, including pre-registration, publication of the review protocol and the documentation of the analytic decisions.

## 7 Limitations

First, this review focused on non-clinical populations. Despite this focus, some studies compared the non-clinical group with a clinical one, and in these cases, we tried to summarise the available information separately for each group, where the separate information was available. We recognise that some studies focusing solely on clinical populations were missed in the process. With the field rapidly advancing, future reviews could focus on the use of naturalistic assessments in clinical contexts, especially for populations who might find engaging with statistic, repetitive assessments more difficult (e.g., individuals with ADHD). Second, due to the scope of the current review, results might not generalise to populations with some physical or mental health conditions. Third, the studies included in this review were divided into three categories according to the method used for the inhibitory control task. However, we acknowledge that these categories could further be divided into sub-categories, for example based on the type of inhibitory control the studies were assessing. However, due to the relatively small number of studies included in this review across all categories (n = 64), we decided that this would not be as informative as evaluating the studies based on the methodology used. Fourth, the current review focused on naturalistic, digital naturalistic tasks. Therefore, tasks using analog naturalistic tasks were not summarised here (e.g., Béraud-Peigné et al., 2023). Nonetheless, we recognise that these tasks have their merits for cognitive assessment and can be suitable for certain populations.

## 8 Conclusions

Naturalistic cognitive research is an emerging field. In this review, we systematically reviewed gamified, virtual reality, and ecological momentary assessment tasks of inhibitory control across the lifespan. We observed high heterogeneity in the types of tasks used, and in the psychometric details reported. Nonetheless, across all categories, we found that naturalistic tasks were largely comparable in terms of performance with standardised equivalents, and that participants generally found these tasks to be as or more enjoyable than computerised tasks using static, decontextualised stimuli. Starting from these results, we discuss several recommendations for the field of naturalistic cognitive research. Specifically, it is essential that the convergent and discriminant validity of naturalistic assessments of inhibitory control, and cognition more generally, is established, and that their feasibility and acceptability are tested. With the emergence of data collection via handheld electronic devices, it is crucial that the test-retest reliability of these ecological momentary cognitive tests is assessed. We also emphasise the importance of collaboration, as naturalistic assessments draw, by design, on the expertise of interdisciplinary teams. Finally, the potential applications of naturalistic cognitive tasks need to be extended to other cognitive domains and populations too, including age groups that have been overlooked by the studies included in this review, notably adolescence, a period of rapid brain development, and patient populations.

## Ethical approval

The study summarised data from published studies and did not require ethical approval.

## Author contributions

LMD, EJD and TUH designed the project. LMD and EJD led and coordinated the project. All authors contributed to the procedures used in the current study. LMD and EJD contributed to screening and data extraction. LMD wrote the first draft of the manuscript. All authors read, edited, and approved the final version of the manuscript.

## Funding

This work was supported by the 10.13039/501100000268Biotechnology and Biological Sciences Research Council [grant number BB/T008709/1].

## Declaration of Competing Interest

The authors have no conflicts of interest to declare.

## Data Availability

https://osf.io/zshkg/

## Footnotes

Appendix A Supplementary data associated with this article can be found in the online version at doi:10.1016/j.neubiorev.2024.105915.
