# Supplementary material for: Naturalistic assessments across the lifespan: Systematic review of inhibition measures in ecological settings
Source: Neurosci Biobehav Rev. 2024 Dec;167:None. doi: 10.1016/j.neubiorev.2024.105915 (PMC11870848; doi:10.1016/j.neubiorev.2024.105915)
Supplement: Supplementary file 1 — Supplementary material. [file mmc1.docx]

**Search strategy Pubmed**

For population, the search terms used were child*[tiab] OR adult*[tiab] OR adolescent[tiab] OR elder*[tiab] OR older[tiab]; for methods, the search terms were virtual[tiab] OR "virtual reality"[tiab] OR "naturalistic"[tiab] OR "ecological momentary assessment"[tiab] OR "ecological sampling method"[tiab] OR ecologic*[tiab] OR "game"[tiab] OR "gamified"[tiab] OR "NIRS"[tiab] OR "near infrared spectroscopy"[tiab] OR "fNIRS"[tiab] OR "EEG"[tiab] OR "electroencephalogram"[tiab] OR “eye movement”[tiab] OR eye-tracking[tiab] OR “motion capture”[tiab] OR “mo-cap”[tiab] OR mocaptiab] OR motion-capture[tiab] OR “magnetoencephalography”[tiab] OR “MEG”[tiab] OR film[tiab] OR movie[tiab] OR robot*[tiab] OR ("real world"[tiab] OR "real life"[tiab]; finally, the search terms for the outcome of interest were "inhibitory control"[tiab] OR "response inhibition"[tiab] OR “inhibition”[tiab]. For the final search, the search terms for population, methods and outcome were combined using the Boolean operator ‘AND’.

**Table S1**. Quality of included studies using gamified and virtual reality tasks.

|  |  | | **Gamified (*N*=23)** | | **Virtual reality (*N*=30)** |  |
| --- | --- | --- | --- | --- | --- | --- |
| **Introduction** |  | |  | |  | |
| **Clear aims/objectives** | |  | |  |  | |
| Yes | |  | | 23 (100%) | 29 (97%) | |
| Don’t know | |  | | 0 (0%) | 0 (0%) | |
| No | |  | | 0 (0%) | 1 (3%) | |
| **Methods** | |  | |  |  | |
| **Appropriate design** | |  | |  |  | |
| Yes | |  | | 22 (96%) | 30 (100%) | |
| Maybe | |  | | 1 (4%) | 0 (0%) | |
| No | |  | | 0 (0%) | 0 (0%) | |
| **Sample size justification** | |  | |  |  | |
| Yes | |  | | 6 (26%) | 1 (3%) | |
| Maybe | |  | | 0 (0%) | 0 (0%) | |
| No | |  | | 17 (74%) | 29 (97%) | |
| **Population clearly defined** | |  | |  |  | |
| Yes | |  | | 23 (100%) | 29 (97%) | |
| Maybe | |  | | 0 (0%) | 0 (0%) | |
| No | |  | | 0 (0%) | 1 (3%) | |
| **Sample taken from appropriate population** | |  | |  |  | |
| Yes | |  | | 20 (87%) | 26 (87%) | |
| Maybe | |  | | 3 (13%) | 4 (13%) | |
| No | |  | | 1 (4%) | 0 (0%) | |
| **Selection process likely to select representative participants** | |  | |  |  | |
| Yes | |  | | 20 (87%) | 26 (87%) | |
| Maybe | |  | | 2 (9%) | 4 (13%) | |
| No | |  | | 1 (4%) | 0 (0%) | |
| **Non-responders (addressed & categorized)** | |  | |  |  | |
| Yes | |  | | 2 (9%) | 0 (0%) | |
| Maybe | |  | | 20 (87%) | 30 (100%) | |
| No | |  | | 1 (4%) | 0 (0%) | |
| **Outcome measured appropriately** | |  | |  |  | |
| Yes | |  | | 23 (100%) | 30 (100%) | |
| Maybe | |  | | 0 (0%) | 0 (0%) | |
| No | |  | | 0 (0%) | 0 (0%) | |
| **Outcome tested using piloted/validated measures** | |  | |  |  | |
| Yes | |  | | 8 (35%) | 21 (70%) | |
| Maybe | |  | | 1 (4%) | 0 (0%) | |
| No | |  | | 14 (61%) | 9 (30%) | |
| **Clear indicators of statistical significance** | |  | |  |  | |
| Yes | |  | | 23 (100%) | 30 (100%) | |
| Maybe | |  | | 0 (0%) | 0 (0%) | |
| No | |  | | 0 (0%) | 0 (0%) | |
| **Methods described in sufficient detail to be repeated** | |  | |  |  | |
| Yes | |  | | 23 (100%) | 29 (97%) | |
| Maybe | |  | | 0 (0%) | 0 (0%) | |
| No | |  | | 0 (0%) | 1 (3%) | |
| **Results** | |  | |  |  | |
| **Basic data described** | |  | |  |  | |
| Yes | |  | | 21 (91%) | 28 (93%) | |
| Maybe | |  | | 0 (0%) | 0 (0%) | |
| No | |  | | 2 (9%) | 2 (7%) | |
| **Concerns about non-response bias** | |  | |  |  | |
| Yes | |  | | 1 (4%) | 0 (0%) | |
| Maybe | |  | | 0 (0%) | 3 (10%) | |
| No | |  | | 22 (96%) | 27 (90%) | |
| **Non-responders were described** | |  | |  |  | |
| Yes | |  | | 11 (48%) | 10 (33%) | |
| Maybe | |  | | 0 (0%) | 0 (0%) | |
| No | |  | | 12 (52%) | 20 (67%) | |
| **Results internally consistent** | |  | |  |  | |
| Yes | |  | | 23 (100%) | 30 (100%) | |
| Maybe | |  | | 0 (0%) | 0 (0%) | |
| No | |  | | 0 (0%) | 0 (0%) | |
| **Results presented for all analyses described in methods** | |  | |  |  | |
| Yes | |  | | 23 (100%) | 30 (100%) | |
| Maybe | |  | | 0 (0%) | 0 (0%) | |
| No | |  | | 0 (0%) | 0 (0%) | |
| **Discussion** | |  | |  |  | |
| **Discussion justified by results** | |  | |  |  | |
| Yes | |  | | 23 (100%) | 30 (100%) | |
| Maybe | |  | | 0 (0%) | 0 (0%) | |
| No | |  | | 0 (0%) | 0 (0%) | |
| **Limitations discussed** | |  | |  |  | |
| Yes | |  | | 21 (91%) | 28 (93%) | |
| Maybe | |  | | 0 (0%) | 0 (0%) | |
| No | |  | | 2 (9%) | 2 (7%) | |
| **Other** | |  | |  |  | |
| **Funding or COIs that could affect interpretation of results** | |  | |  |  | |
| Yes | |  | | 0 (0%) | 0 (0%) | |
| Maybe | |  | | 0 (0%) | 0 (0%) | |
| No | |  | | 23 (100%) | 30 (100%) | |
| **Ethical approval or consent obtained** | |  | |  |  | |
| Yes | |  | | 23 (100%) | 30 (100%) | |
| Maybe | |  | | 0 (0%) | 0 (0%) | |
| No | |  | | 0 (0%) | 0 (0%) | |

**Table S2***.* Quality of included EMA studies.

|  | **Overall (N=12)** |
| --- | --- |
| **Quality 1 – Provided rationale for the EMA design** |  |
| Weak | 0 (0%) |
| Moderate | 0 (0%) |
| Strong | 12 (100%) |
| **Quality 2 – Conducted a priori power analysis** |  |
| Weak | 7 (58%) |
| Moderate | 3 (25%) |
| Strong | 2 (17%) |
| **Quality 3 – Adherence to the EMA protocol** |  |
| Weak | 5 (42%) |
| Moderate | 0 (0%) |
| Strong | 7 (58%) |
| **Quality 4 – Treatment of missingness** |  |
| Weak | 9 (75%) |
| Moderate | 0 (0%) |
| Strong | 3 (25%) |

**Table S3.** Studies included in the systematic review by methodological category.

| **Category** | **No.** | **Title** | **Authors and year** | **Task name** |
| --- | --- | --- | --- | --- |
| **Gamification** | 1 | Scaffolding executive function capabilities via play-&-learn software for preschoolers | Axelsson et al. (2016) | Bird Hero |
|  | 2 | Development, feasibility and acceptability of a gamified cognitive DEvelopmental assessment on an E-Platform (DEEP) in rural Indian pre-schoolers - a pilot study | Bhavnani et al. (2019) | Grow Your Garden |
|  | 3 | FarmApp: A new assessment of cognitive control and memory for children and young people with neurodevelopmental difficulties | Brkic et al. (2022) | Sheep Game |
|  | 4 | Antonyms: A Computer Game to Improve Inhibitory Control of Impulsivity in Children with Attention Deficit/Hyperactivity Disorder (ADHD) | Crepaldi et al. (2020a) | Central Building |
|  | 5 | The Use of a Serious Game to Assess Inhibition Mechanisms in Children | Crepaldi et al. (2020b) | Central Building |
|  | 6 | Validation of new online game-based executive function tasks for children | Johann & Karbach (2018) | Go/No-Go, Flanker, Stroop gamified tasks |
|  | 7 | ADHD outside the laboratory: Boys' executive function performance on tasks in videogame play and on a visit to the zoo | Lawrence et al. (2002) | Crash Bandicoot |
|  | 8 | A Study on the Validity of a Computer-Based Game to Assess Cognitive Processes, Reward Mechanisms, and Time Perception in Children Aged 4-8 Years | Peijnenborgh et al. (2016) | Timo's Adventure |
|  | 9 | The Effects of Reward And Experience Valence In A Videogame-Task Designed To Evaluate Response Inhibition | Rivero et al. (2021) | Dragon Hunter Task |
|  | 10 | Using a serious game to measure executive functioning: Response inhibition ability | Tong et al. (2021) | Whack-a-mole |
|  | 11 | FISHERMAN: A Serious Game for Executive Function Assessment of Older Adults | Wang et al. (2023) | Cautious Fisherman |
|  | 12 | Effective Gamification of the Stop-Signal Task: Two Controlled Laboratory Experiments | Friehs et al. (2020) | Stop Signal Task |
|  | 13 | Attrition from Web-Based Cognitive Testing: A Repeated Measures Comparison of Gamification Techniques | Lumsden et al. (2017) | Stop Signal Task |
|  | 14 | Preserved Inhibitory Control Deficits of Overweight Participants in a Gamified Stop-Signal Task: Experimental Study of Validity | Schroeder et al. (2021) | Stop Signal Task |
|  | 15 | A proof-of-concept study exploring the effects of impulsivity on a gamified version of the stop-signal task in children | Gallagher et al. (2023) | Stop Signal Task |
|  | 16 | Proactive and Reactive Response Inhibition across the Lifespan | Smittenaar et al. (2015) | Fruit tap |
|  | 17 | TENI: A comprehensive battery for cognitive assessment based on games and technology | Delgado et al. (2016) | BZZ!INH |
|  | 18 | More than skin deep: about the influence of self-relevant avatars on inhibitory control | Friehs et al. (2022) | Stop Signal Task |
|  | 19 | Shocking advantage! Improving digital game performance using non-invasive brain stimulation | Friehs et al. (2021) | Stop Signal Task |
|  | 20 | Are 3D virtual environments better than 2D interfaces in serious games performance? An explorative study for the assessment of executive functions | Chicchi et al. (2021) | Go/No-Go (AT3) and Stroop (AT4) |
|  | 21 | EXPANSE: A novel narrative serious game for the behavioural assessment of cognitive abilities | Chicchi et al. (2018) | Go/No-Go (AT3) and Stroop (AT4) |
|  | 22 | The Long and Winding Road to Real-Life Experiments: Remote Assessment of Executive Functions with Computerized Games-Results from 8 Years of Naturalistic Interventions | Vladisauskas et al. (2024) | Stroop (Heart–Flower Stroop Test) and Flanker (The Child Attentional Networks Task) |
|  | 23 | Executive functions and classroom behaviour in second graders | Heemskerk & Roebers (2023) | Stroop (Heart–Flower Stroop Test) |
| **Virtual reality** | 1 | Virtual reality's effect on children's inhibitory control, social compliance, and sharing | Bailey et al. (2019) | Simon Says |
|  | 2 | Attentional profile of adolescents with ADHD in virtual-reality dual execution tasks: A pilot study | Camacho-Conde & Climent (2022) | Nesplora Aquarium |
|  | 3 | Development and initial assessment of a new paradigm for assessing cognitive and motor inhibition: the bimodal virtual-reality Stroop | Henry et al. (2012) | VR Stroop |
|  | 4 | Effect of Distractors on Sustained Attention and Hyperactivity in Youth With Attention Deficit Hyperactivity Disorder Using a Mobile Virtual Reality School Program | Hong et al. (2022) | VR rapid visual information processing task |
|  | 5 | Assessment of executive function in adolescence: a comparison of traditional and virtual reality tools | Lalonde et al. (2013) | ClinicaVR: Classroom-Stroop |
|  | 6 | Using virtual reality to define the mechanisms linking symptoms with cognitive deficits in attention deficit hyperactivity disorder | Mangalmurti et al. (2020) | ClinicalVR: Classroom CPT |
|  | 7 | The Influence of Methylphenidate on Hyperactivity and Attention Deficits in Children With ADHD: A Virtual Classroom Test | Muhlberger et al. (2020) | ClinicalVR: Classroom CPT |
|  | 8 | Virtual reality as a screening tool for sports concussion in adolescents | Nolin et al. (2012) | ClinicalVR: Classroom CPT |
|  | 9 | ClinicaVR: Classroom-CPT: A virtual reality tool for assessing attention and inhibition in children and adolescents | Nolin et al. (2016) | ClinicalVR: Classroom CPT |
|  | 10 | Virtual Apartment-Based Stroop for assessing distractor inhibition in healthy aging | Parsons & Barnett (2019) | VR Stroop |
|  | 11 | Virtual apartment stroop task: Comparison with computerized and traditional stroop tasks | Parsons & Barnett (2018) | VR Stroop |
|  | 12 | Usability and validity of a virtual reality cognitive assessment tool for pediatric traumatic brain injury | Shen et al. (2022) | VR Stroop |
|  | 13 | New virtual reality tool (Nesplora Aquarium) for assessing attention and working memory in adults: A normative study | Climent et al. (2021) | Nesplora Aquarium |
|  | 14 | Analysis of cognitive and attentional profiles in children with and without ADHD using an innovative virtual reality tool | Areces et al. (2018) | Nesplora Aquarium |
|  | 15 | Development and Preliminary Validation of a Virtual Reality–Based Measure of Response Inhibition Under Normal and Stressful Conditions | Donahue & Shrestha (2019) | VR Stroop |
|  | 16 | Presence and simulator sickness predict the usability of a virtual reality attention task | Voinescu, Petrini & Fraser (2023) | Nesplora Aquarium |
|  | 17 | The effectiveness of a virtual reality attention task to predict depression and anxiety in comparison with current clinical measures | Voinescu et al. (2023) | Nesplora Aquarium |
|  | 18 | Virtual-reality-based attention assessment of ADHD: ClinicaVR: Classroom-CPT versus a traditional continuous performance test | Negut, Jurma & David (2016) | ClinicaVR: Classroom-CPT |
|  | 19 | Distractibility In Attention/Deficit/ Hyperactivity Disorder (ADHD): The Virtual Reality Classroom | Adams et al. (2009) | VR CPT |
|  | 20 | A Controlled Clinical Comparison of Attention Performance in Children with ADHD in a Virtual Reality Classroom Compared to Standard Neuropsychological Methods | Parsons et al. (2007) | VR CPT |
|  | 21 | Is a Virtual Reality Test Able to Predict Current and Retrospective ADHD Symptoms in Adulthood and Adolescence? | Areces et al. (2019) | Nesplora Aquarium |
|  | 22 | Comparison between two continuous performance tests for identifying ADHD: Traditional vs. virtual reality | Rodriguez et al. (2018) | Nesplora Aquarium |
|  | 23 | AULA—Advanced Virtual Reality Tool for the Assessment of Attention: Normative Study in Spain | Iriarte et al. (2016) | Nesplora Aquarium |
|  | 24 | Multimodal Virtual Reality-Based Assessment of Adult ADHD: A Feasibility Study in Healthy Subjects | Wiebe et al. (2023) | Virtual Seminar Room |
|  | 25 | Bimodal virtual reality Stroop for assessing distractor inhibition in autism spectrum disorders | Parsons & Carlew (2016) | VR Classroom Bimodal Stroop |
|  | 26 | Virtual reality Stroop task for assessment of supervisory attentional processing | Parsons, Courtney & Dawson (2013) | VR Stroop |
|  | 27 | Are 3D virtual environments better than 2D interfaces in serious games performance? An explorative study for the assessment of executive functions | Chicchi et al. (2019) | Go/No-Go (AT3) and Stroop (AT4) |
|  | 28 | Neuronal Correlates of Task Irrelevant Distractions Enhance the Detection of Attention Deficit/Hyperactivity Disorder | Chen et al. (2023) | VR CPT |
|  | 29 | Data-driven profiles of attention-deficit/hyperactivity disorder using objective and ecological measures of attention, distractibility, and hyperactivity | Fernández-Martín et al. (2024) | VR CPT |
|  | 30 | Oscillatory Neural Correlates of Police Firearms Decision-Making in Virtual Reality | Alexander et al. (2024) | VR CPT |
| **EMA** | 1 | Does real time variability in inhibitory control drive snacking behavior? An intensive longitudinal study | Powell et al. (2017) | Go/No-Go |
|  | 2 | The Digital Marshmallow Test (DMT) Diagnostic and Monitoring Mobile Health App for Impulsive Behavior: Development and Validation Study | Sobolev et al. (2021) | mGNG |
|  | 3 | The feasibility and acceptability of assessing inhibitory control and working memory among adolescents via an ecological momentary assessment approach | Warren & Pentz (2019) | Flanker |
|  | 4 | Using Smartphone Sensor Data to Assess Inhibitory Control in the Wild: Longitudinal Study | Tseng et al. (2020) | Stop signal task |
|  | 5 | Inhibitory control and mood in relation to psychological resilience: an ecological momentary assessment study | Nahum et al. (2023) | CPT |
|  | 6 | Less inhibited and more depressed? The puzzling association between mood, inhibitory control and depressive symptoms | Yitzhak et al. (2023) | CPT |
|  | 7 | Everyday Cognitive Control and Emotion Dysregulation in Young Adults With and Without ADHD: An Ecological Momentary Assessment Study | Ben-Dor Cohen et al. (2023) | CPT |
|  | 8 | 100 days of Adolescence: Elucidating Externalizing Behaviors Through the Daily Assessment of Inhibitory Control | Chaku et al. (2023) | Stroop |
|  | 9 | Multi-level prediction of substance use: Interaction of white matter integrity, resting-state connectivity and inhibitory control measured repeatedly in every-day life | Chirokoff et al. (2024) | Stroop |
|  | 10 | Identifying the role of (dis)inhibition in the vicious cycle of substance use through ecological momentary assessment and resting-state fMRI | Chirokoff et al. (2024) | Stroop |
|  | 11 | Extended ambulatory assessment of executive function: within-person reliability of working memory and inhibitory control tasks | Dali et al. (2024) | SST |
|  | 12 | Brief Report: Feasibility and Validity of Mobile Cognitive Testing in Patients With Substance Use Disorders and Healthy Controls | Bouvard et al. (2018) | Stroop |
